# Wharton’s Jelly-Derived Mesenchymal Stem Cells: Phenotypic Characterization and Optimizing Their Therapeutic Potential for Clinical Applications

**DOI:** 10.3390/ijms140611692

**Published:** 2013-05-31

**Authors:** Dae-Won Kim, Meaghan Staples, Kazutaka Shinozuka, Paolina Pantcheva, Sung-Don Kang, Cesar V. Borlongan

**Affiliations:** 1 Department of Neurosurgery, Institute of Wonkwang Medical Science, School of Medicine, Wonkwang University, 344-2 Shinyong-dong, Iksan 570-749, Korea; E-Mails: kimdw@wku.ac.kr (D-W.K.); kangsd@wku.ac.kr (S-D.K.); 2 Center of Excellence for Aging and Brain Repair, Department of Neurosurgery and Brain Repair, University of South Florida College of Medicine, Tampa, FL 33612, USA; E-Mails: meaghans@mail.usf.edu (M.S.); kshinozu@health.usf.edu (K.S.); ppantche@mail.usf.edu (P.P.)

**Keywords:** umbilical cord, wharton’s jelly, mesenchymal stem cells, phenotypic characteristics, therapeutic applications, experimental protocol

## Abstract

Wharton’s jelly (WJ) is a gelatinous tissue within the umbilical cord that contains myofibroblast-like stromal cells. A unique cell population of WJ that has been suggested as displaying the stemness phenotype is the mesenchymal stromal cells (MSCs). Because MSCs’ stemness and immune properties appear to be more robustly expressed and functional which are more comparable with fetal than adult-derived MSCs, MSCs harvested from the “young” WJ are considered much more proliferative, immunosuppressive, and even therapeutically active stem cells than those isolated from older, adult tissue sources such as the bone marrow or adipose. The present review discusses the phenotypic characteristics, therapeutic applications, and optimization of experimental protocols for WJ-derived stem cells. MSCs derived from WJ display promising transplantable features, including ease of sourcing, *in vitro* expandability, differentiation abilities, immune-evasion and immune-regulation capacities. Accumulating evidence demonstrates that WJ-derived stem cells possess many potential advantages as transplantable cells for treatment of various diseases (e.g., cancer, chronic liver disease, cardiovascular diseases, nerve, cartilage and tendon injury). Additional studies are warranted to translate the use of WJ-derived stem cells for clinical applications.

## 1. Introduction

The advent of stem cells as a tool to decipher the cell’s biology and as a source of transplant therapy to correct aging and diseases has become a core research arena for tissue engineering and regenerative medicine. A pivotal source of stem cells is the umbilical cord’s Wharton’s jelly (WJ) [1]. A unique cell population of WJ that has been suggested as displaying the stemness phenotype is the mesenchymal stromal cells or MSCs. The prototypical feature of MSCs is their plastic adherence expressing a phenotypically defined set of surface markers including CD90, CD73 and CD105. Although MSCs have been harvested from many different tissues, novel considerations of tissue specificity may dictate the eventual fate of MSCs. In particular, MSCs’ stemness and immune properties appear to be more robustly expressed and functional with fetal than adult-derived MSCs. To this end, the young age of WJ suggests that MSCs harvested from this fetal origin will exhibit a much more proliferative, immunosuppressive, and even therapeutically active stem cells than those isolated from older, adult tissue sources such as the bone marrow or adipose. This alternative source of MSCs became feasible with the report by McElreavey *et al*. [2] of the culture of cells from WJ, which is the primitive connective tissue of the human umbilical cord (UC), first described by Thomas Wharton in 1656 [3]. Thereafter, research efforts have attempted to optimize the isolation and differentiation of these cells derived from WJ [4–11]. The present compilation of milestone discoveries on WJ-derived stem cells should aid in further moving the field of cell biology and therapy towards clinical applications.

## 2. Anatomical Relationship of Various UC Structures and WJ as Sources of MSCs

**Figure 1:**
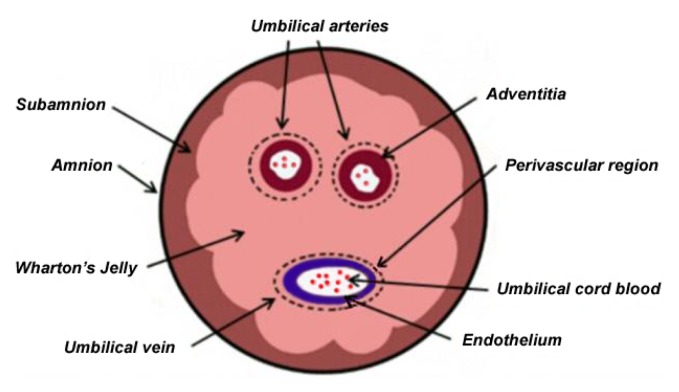
Cross-sectional diagram of human umbilical cord shows anatomical compartments, including Wharton’s jelly, as a source of stem cells.

During pregnancy, the fetus and placenta is connected by an elastic UC which prevents umbilical vessels from compression, torsion, and bending while providing a good blood circulation. Anatomically, the UC consists of two umbilical arteries and one umbilical vein, both embedded within a specific mucous proteoglycan-rich matrix, known as WJ, which is then covered by amniotic epithelium (Figure 1).

WJ which contains a multipotent fibroblast-like MSC population were first obtained more than 10 years ago [12]. Previously, WJ-MSCs were termed as “umbilical cord matrix stem cells (UCMSCs)” to distinguish them from endothelial cells isolated from umbilical vein (HUVEC) as well as MSCs isolated from UC blood (UCB-MSCs) [13,14].

There are two possible theories on how stem cells existed in the WJ. First, there were two waves of migration of fetal MSCs in early human development. During these waves of migration, some of MSCs got trapped and resided in the gelatinous WJ of the UC [15]. Second, the cells in the WJ are actually primitive MSCs originating from mesenchyme that were already there within the UC matrix. The function of these cells may be to secrete the various glycoproteins, mucopolysaccharides, glycosaminoglycans and extracellular matrix proteins to form a gelatinous ground substance to prevent strangulation of the UC vessels during gestation [16].

Stem cells have been derived in the amniotic compartment (outer epithelial layer and inner subamniotic mesenchymal layer), the WJ compartment, the perivascular compartment surrounding the vessels, the media and adventitia compartment of the walls of UC blood vessels, the endothelial compartment (inner lining of the vein) and the vascular compartment (blood lying within the UC blood vessels) [16]. All these compartments have been described as distinct regions [17] and the nomenclature has not been standardized, with terms such as “subamnion”, “cord lining (sub-amnio)”, “intervascular”, “perivascular” and “hUVEC” being used. Also, isolation methods and region of interest for WJ-MSCs have not been standardized. Indeed, it is not known whether the stem cell populations within WJ-MSCs between compartments are one and the same as there is no clear demarcation histologically between these compartments. At the same time the various individual derivation protocols are ambiguous and further compound the differences in stem cell populations between compartments [16]. WJ-MSCs can be isolated from two regions, namely, intervascular and sub-amnion [18], while others have isolated WJ-MSCs from three regions, namely, the perivascular zone, the inter-vascular zone, and the sub-amnion [19]. Structural, immunohistochemical, and functional analysis performed *in vitro* show significant differences in the number and nature of cells among these three regions and they have different properties [20,21]. These findings led to the hypothesis that these regions might be originating from different pre-existing structures [22]. A stem cell population has been isolated from around the umbilical vessels, termed human umbilical cord perivascular cells (HUCPVCs) [23,24] while equally potent stem cell-like cells have been harvested from sub-amnion (cord lining; CL) [17,25]. Of note, WJ-MSCs located close to amniotic surface display enhanced ability to proliferate, whereas WJ-MSCs with more differentiated were found in closer proximity to the umbilical vessels [20,21].

## 3. Characteristic Features of WJ-MSCs for Cell Therapy

### 3.1. Sources of Stem Cells

Various types of stem cells have been isolated to date in the human from a variety of tissues including preimplantation embryos, fetuses, birth-associated tissues and adult organs. Based on biochemical and genomic markers, they can be broadly classified into embryonic stem cells (ESC), mesenchymal stem cells (MSC), and hematopoietic stem cells (HPS).

ESCs are pluripotent stem cells which theoretically can be differentiated into almost all tissues in the human body. However, ESCs have limitation for use. The principal limitation is an ethical problem. Because ESCs are derived from the inner cell mass of a blastocyst, an early-stage embryo [26], isolating the embryoblast or inner cell mass results in destruction of the fertilized human embryo, which raises ethical issues. Although the source of the blastocyst was generally discarded material from *in vitro* fertilization clinics there is no consensus whether or not a human life at the embryonic stage should be granted the moral status of a human being [27]. Other limitations are the risks of immunorejection and tumorigenesis. To overcome the problem of immunorejection, protocols were developed where tissue could be personalized to patients by transfecting the patient’s somatic cells with pluripotent genes to produce human induced pluripotent stem cells (hiPSCs); unfortunately, epigenetic changes in the form of chromosomal duplications and deletions have been reported in the ensuing hiPSCs [28,29]. Additionally, hiPSCs induce tumorigenesis in immunodeficient mice and such teratoma formation is faster and more efficient than their ESCs counterpart [30]. The risk of tumorigenesis is of particular importance when using pluripotent cells, since these are characterized by the ability to form teratomas in animal models [26,29]. Thus, the differentiation state of transplanted cells will need to be defined with high precision to avoid delivery of residual pluripotent cells that may differentiate aberrantly *in vivo*.

HSCs have limited plasticity in that they can differentiate only into blood and blood-related lineages. In addition, the HSC numbers from bone marrow and UC are low and require *ex vivo* expansion for the treatment hematologic diseases in adult humans. However, a recent study showed there is strong evidence that HSCs are pluripotent and are the source for the majority, if not all, of the cell types in our body [31].

Fetal MSCs are controversial as they are derived from human abortuses. Since Pittenger and colleagues demonstrated the successful isolation of multipotent MSCs from bone marrow, it has become the primary source from which to obtain MSCs [32]. Although BM-MSCs are the most studied and well-documented, BM-MSCs have limitation in terms of cell numbers and as such require expansion *in vitro* running the risk of loss of stemness properties, induction of artifactual chromosomal changes, and problems of contamination [16,32]. Adipose tissue has recently emerged as an alternative source of MSCs. Despite its plentiful nature, an invasive procedure is still required to collect the tissue [33].

Extra-embryonic perinatal MSCs harvested from placenta, fetal membrane (amnion and chorion), UC, UC blood, and amniotic fluid represent an intermediate stem cell type that partially combines some pluripotent properties of adult MSCs [34–37]. Because they have close ontogenetic relationship with embryonic stem cells, extra-embryonic tissue-derived MSCs have immunoprivileged characteristics, possess a broader multipotent plasticity, and proliferate faster than adult MSCs [37,38]. Moreover, these cells could be isolated and used without ethical problem, because extra-embryonic tissues are normally discarded after birth [38].

### 3.2. Immunomodulatory Property of WJ

The practical utility of WJ-MSCs would be in allogeneic transplantation. One important requisite for allogeneic transplantation is low immunogenicity. The therapeutic utility of the WJ-derived stem cells can be ascribed to their regenerative and immunomodulatory potential of these cells. A review paper discusses immunomodulatory molecules expressed by WJ-MSC and also analyzes the *in vitro* and *in vivo* data on their immune-modulating activities [18]. WJ-MSCs are also capable of immune suppression and immune avoidance similar to other types of MSCs. They express MHC class I (HLA-ABC) at low levels but not class II (HLA-DR) and co-stimulatory antigens such as CD80, CD86 implicated in activation of both T and B cell responses [18,39–42]. Low levels of MHC class I antigens could be a mechanism to protect them from Natural killer cell-mediated lysis [18]. Even though the overall expression of immune-stimulatory ligands on WJ-MSCs remains similar to that of bone marrow-derived MSCs (BM-MSCs), their induction with pro-inflammatory cytokines might differ. HLA-DR is induced substantially in BM-MSCs with IFN-γ treatment but the induction is very negligible in WJ-MSCs [39,43]. In addition, WJ-MSCs produce large amounts of tolerogenic IL-10, higher levels of TGF-β than BM-MSCs, and express HLA-G, which is not expressed in BM-MSCs [39,40,42,43]. HLA-G appears to play a role in the immune tolerance during pregnancy by evading a maternal immune response against the fetus and inducing the expansion of regulatory T cells, which would contribute to the suppression of effectors responses to alloantigens [44,45]. Compelling evidence has shown that the low rate of rejection seems to be associated to the expression of these antigens in blood, heart and liver/kidney grafts [46]. Furthermore, WJ-MSCs express IL-6 and VEGF, which have recently been shown to be pivotal in the immunosuppressive capability of MSCs [42,47]. WJ-MSCs are less immunogenic than BMMSCs as well as fetal MSCs making them more amenable for allogeneic as well as xenogeneic transplantation. However, under certain circumstances, UCMSCs can elicit an immune response. A single injection of MHC mismatched inactivated UCMSCs did not induce a detectable immune response. When injected in an inflamed region, injected repeatedly in the same region, or stimulated with IFN-γ prior to injection, UCMSCs can be immunogenic [48]. Therefore, care must be taken to avoid sensitization against the cell therapy, especially if these cells are used for repairing damaged, inflamed tissue that needs repeated administration into the same location.

WJ-MSCs also afford robust immunomodulatory properties compared to BM-MSCs. BM-MSCs have been widely reported to attenuate mitogen driven as well as alloantigen or specific antigen driven T cell response in a dose dependent manner *in vitro* [49]. MSCs have been shown to equally inhibit CD4(+), CD8(+), CD2(+) and CD3(+) subsets [50]. However, WJ-MSCs exhibit a prominent suppression even at very low dose range as compared to BM-MSCs in terms of mitogen induced CD3(+) T cell responses [39,51]. In addition, WJ-MSCs suppress allogeneically-stimulated T cells to a greater extent than either BM-MSCs or adipose-derived MSCs [18]. Fetal liver-derived MSCs suppress lympho-proliferative responses to mitogens, but do not attenuate allo-proliferative responses [52]. In this context, peri-natal MSCs, like that of WJ-MSCs, not only seem to attenuate lymphoproliferation more robustly than BM-MSCs, but also the regulation is stimuli-independent unlike fetal MSCs [18]. Additionally, WJ-MSCs can affect the maturation and activation of dendritic cell (DC) precursors. WJ-MSCs, when cultured with CD14(+) monocytes, inhibited their differentiation into mature DCs in a contact-dependent manner. WJ-MSCs co-cultured monocytes were shown to be locked in an immature DC phenotype and the up-regulation of co-stimulatory ligands was blocked in the co-cultures [53]. Thus, WJ-MSCs might indirectly affect T cell allogeneic responses through attenuation of DC functions. There are a limited number of studies with purified populations of immune cells tracing their activation and effector functions closely in presence of WJ-MSCs. Prasanna *et al*. have tracked the pro-inflammatory cytokine secretion patterns kinetically in co-cultures of WJ-MSCs/BM-MSCs with PHA-activated lymphocytes [39]. A change in the threshold and kinetics of IL-2 secretion was observed only with BM-MSCs and not with WJ-MSCs. Additionally, an early activation of negative co-stimulatory ligands on peripheral blood lymphocytes was observed more evidently with WJ-MSCs co-cultures [39]. Although the major secretary profiles of different tissue derived MSCs are similar, WJ-MSCs and cord blood MSCs only secrete IL-12, IL-15 and Platelet-derived growth factor (PDGF). In summary, the putative mechanisms of immunomodulatory properties of WJ-MSCs include upregulation of negative co-stimulatory ligands, secretion of immunosuppressive soluble factors, generation of memory cells, cell fusion to escape recognition, immune avoidance mechanisms specific to fetal-maternal interface, attenuation of antigen-presenting cell functions, altered migration of immune cells, and T cell anergy apoptosis tolerance [18].

### 3.3. Phenotypic Characterization of WJ

In 2011, Conconi *et al*., laid out the groundwork on the WJ’s characterization by providing an overview on the human UC [54]. In this review, a panoramic view of phenotypic characteristics of human UC cells derived from various UC parts are described. The high heterogeneity of extraction, culture, and analysis procedures hinder the ability to precisely identify UC stromal cells. Overall, cells from WJ fit with the minimal criteria for MSCs. The mesenchymal features of WJ cells have been confirmed by the expression of specific lineage cytoskeletal markers, such as SMA and vimentin. Furthermore, ESC markers, such as Oct-4, SSEA4, nucleostemin, SOX-2 and Nanog, have also been revealed, though HUCPV cells do not express Oct-4, SSEA4. Other cell surface molecules are CD59 and CD146 which are not expressed in HUCPV cells. CD59 is involved in the complement system regulation thus preventing cell lysis. CD146 is a cell adhesion molecule expressed not only on endothelial cells but also on MSCs[54]. Furthermore, the HUCPV cells stain for pan-cytokeratin more strongly than WJ-MSCs [20]. This group suggested that HUCPV cells are more differentiated than WJ-MSCs and this explains why the HUCPV cells may not differentiate to neuronal cells. The most outstanding feature of CL-MSCs is the expression of CD14 which is not expressed in WJ-MSCs [25]. CD14 is widely recognized as a common marker for marcrophages. The function and significance of CD14 expression on CL-MSCs has not to be determined yet, but it is interesting to note that the soluble form of CD14 can down regulate T cell activation [55]. The most striking feature of WJ-MSCs is their unique ability to express the HLA-G6 isoform. As mentioned previously, HLA-G6 is implicated in immune-modulation. Thus, WJ-MSCs are particularly suitable for cell-based therapy. As a result, different phenotypic profiles are detectable not only among the cells obtained from the various parts of cord, but also inside the same UC regions, suggesting that UCMSCs may represent an unique cell family whose components present various degree of stemness. However, *in vitro* and *in vivo* evidence indicates WJ as an excellent source of MSCs because its cells present a wide range of potential therapeutic applications. In addition, Conconi and co-workers [56] first reported that CD105(+)/CD31(−)/KDR(−) cells from WJ are able not only to differentiate *in vivo* towards the myogenic lineage, but also to contribute to the muscle regenerative process. Such myogenic differentiation potential of CD105(+) cells from WJ was further confirmed using *in vitro* assays.

Subsequently, Jeschke and colleagues identified the specific region of the UC lining (sub-amnion) and WJ enriched with stem cell niches [17]. Before this report, Kita and co-workers [25] previously attempted to isolate MSCs from sub-amnion of the UC and they reported that sub-amniotic MSCs are distinct from ESCs and do not show tumorigenicity *in vitro*. The CL-MSCs isolated by their method maintain typical characteristics of MSCs *in vitro*, but also showed several specific features [25]. Because of several anatomically distinct zones found in the UC, isolated multipotent cells sometimes show heterogeneity. In addition, differences in isolation technique may lead to further variation. Of note, CL-MSCs have excellent potential in terms of their proliferative capacity and possibly multipotency [17]. However, the main disadvantage of CL-MSC is the extremely time-consuming nature of the isolation process. In contrast, WJ provides an ample supply of MSCs. Although WJ–MSCs show more variation in terms of quality of cells, WJ is still a very useful depot of MSCs. Accordingly, the choice of MSC source should consider the quality and quantity of stem cells required for each specific application.

Interestingly, biological characteristics of MSCs can be influenced by perinatal environment. There is increasing evidence that intrauterine metabolic disturbances produced by hyperglycemia during pregnancy appear to increase the risk in offspring for obesity and diabetes [57–59]. In addition, studies in animal models suggest that the MSC commitment into pre-adipocytes begins during fetal development and perinatal life [60]. Since the number of pre-adipocytes and mature adipocytes is lower in normal subjects than in obese subjects [61], changes in the prenatal maturational process may play a role in the pathogenesis of obesity and metabolic-associated diseases. For this reason, it would be useful to investigate how the perinatal environment may affect fetus-derived MSCs, especially in unregulated gestational diabetes. Recently, Pierdomenico *et al*., have compared WJ-MSCs obtained from UC of both healthy and diabetic mothers, in order to better understand the mechanisms involved in metabolic diseases in offspring of diabetic mothers [62]. Although the same markers were expressed in WJ-MSCs obtained from both healthy and diabetic mothers, their expression levels differed, possibly due to a difference in functional characteristics of the two WJ-MSCs groups. Lower levels of CD90 were observed in WJ-MSCs from diabetic mothers, which could be to the result of a plasticity decrease of these cells. It was also shown that WJ-MSCs from diabetic mothers presented higher adipocyte differentiation efficiency, compared to WJMSCs obtained from healthy mothers, suggesting, therefore, a possible pre-commitment of these cells to the adipogenic lineage. In addition, the up-regulation of CD44, CD29, CD73, CD166, SSEA4 and TERT in WJ-MSCs obtained from diabetic mothers might be related to the slight increase of proliferative ability of these cells. Results indicate that in contrast to cells from healthy mothers, WJ-MSC from diabetic mothers display a higher ability to differentiate towards the adipogenic lineage. This suggests that the diabetic uterine environment may be responsible for a “pre-commitment” that could give rise in the post natal life to an alteration of adipocyte production upon an incorrect diet style, which in turn would produce obesity.

## 4. Clinical Applications of WJ-Derived Stem Cells

### 4.1. Cancer Therapy

Stem cell based therapy has significant potential to treat various diseases including primary and metastatic cancers. Tamura and co-workers reported previously showed that un-engineered human and rat UCMSC significantly attenuated the growth of multiple cancer cell lines *in vivo* and *in vitro* through multiple mechanisms [63,64]. Intrinsic stem cell-dependent regulation of cancer growth, potential mechanisms involved in this unique biological function, delivery of exogenous anti-cancer agents, and the potential for clinical applications were discussed in a previous paper [65]. Since naive UCMSC have the intrinsic ability to secrete factors that can result in cancer cell growth inhibition and/or apoptosis *in vitro* and *in vivo*, they have many advantages for cell-directed cancer therapy. The mechanisms by which naïve UCMSC attenuate tumor growth have yet to be fully clarified, however, two potential mechanisms have been suggested [65]. The first potential mechanism is production of multiple secretory proteins that induce cell death of cancer cells and cell cycle arrest. This suggests that UCMSC stimulate caspase activities and arrest the cell cycle even in the absence of direct contact with cancer cells [43,66]. In addition, microarray analysis of rat UCMSC revealed over-expression of multiple tumor suppressor gene [65]. The second potential mechanism is the enhancement of an immune reaction to cancer cells. Immunohistochemistry revealed that the majority of infiltrating lymphocytes in rat UCMSC-treated tumors were T cells. The treatment of rat UCMSC apparently increased CD8(+) T cell infiltration throughout the tumor tissue [64]. Although these results contradict results described above which show the low immunogenicity of human UCMSC, the immunogenicity of UCMSC in tumor bearing animals may be dependent upon the microenvironment of UCMSC and tumor cells.

The homing ability of stem cells seems to be mediated by the interaction of cytokines/growth factors and their receptors. Large amounts of various cytokines and growth factors are secreted by tumor cells. Since UCMSC and other MSCs express various cytokine and growth factor receptors on their surface, they are likely to migrate towards cytokine/growth factor production sites by sensing these cytokine gradients [65]. Due to the over-expression of IL-8 receptor and CXCR, UCMSCs have a greater capacity to migrate towards tumor than BM-MSCs. It has also been demonstrated that these cells can be engineered to express cytotoxic cytokines before being delivered to the tumor and can be preloaded with nanoparticle payloads and attenuate tumors after homing to them [67,68]. Human UCMSC engineered to express INF-β produced sufficient amounts of INF-β to induce death of human breast adenocarcinoma cells and bronchioloalveolar carcinoma cells *in vitro* and *in vivo* [41,68]. Thus, the INF-β-human UCMSC could also be a new therapeutic modality for the treatment of various cancers. Among many tissue-originated multipotent stem cells, UCMSC may be suitable for allogenic transplantation as a therapeutic tool due to their abundance, low immunogenicity, lack of CD34 and CD45 expression, and simplicity of the methods for harvest and *in vitro* expansion. The homing ability to inflammatory tissues, including cancer tissues, and tumoricidal ability of UCMSC further confers upon these cells the potential for targeted cancer therapy.

### 4.2. Liver Disease

Cell therapy has also emerged as an attractive alternative to orthotopic liver transplantation for the treatment of liver disease. WJ-MSCs have demonstrated a potential to differentiate into endodermal lineage, including hepatocyte-like cells. The *in vitro* and *in vivo* use of UCMSCs for liver cell therapy has been described [69]. UCMSCs represent a very attractive cell source for treatment of liver disease as they display several hepatic markers characterizing the sequential steps of liver development. Moreover, *in vivo* experiments showed that after transplantation of undifferentiated UCMSCs in the liver of SCID mice with partial hepatectomy, the engrafted cells expressed human hepatic markers such as albumin and AFP, after 2, 4, and 6 weeks following transplantation. This strongly suggests that UCMSCs could be of great interest for the regenerative medicine approaches in liver disease [70]. Interestingly, a different study suggests a supportive role of undifferentiated UCMSCs in rescuing injured liver functions and reducing fibrosis *in vivo*. This study supports the hypothesis that, even in the absence of an actual transdifferentiation process, UCMSCs could exert a supportive action in increasing the functional recovery of recipient livers, perhaps stimulating the differentiation of endogenous parenchymal cells and promoting degradation of fibrous matrix [71]. In addition, their differentiation ability to hepatic lineage can be enhanced *in vivo* and *in vitro* after culture with hepatogenic factors. In treating liver cirrhosis, UCMSCs have properties of anti-inflammatory and anti-fibrosis by endogenous secreted factors such as metalloproteinases. This ability of UCMSCs to differentiate into hepatocyte-like cell warrant further investigations designed to better understand that cells can repopulate and rescue the liver function.

### 4.3. Cardiovascular Diseases

The therapeutic potential of WJ for cardiovascular tissue engineering has been suggested [72]. Because surgical treatment using non-autologous valves or conduits have distinct disadvantages including obstructive tissue ingrowths and calcification of the implant [73,74], cardiovascular fetal tissue engineering focuses on the *in vitro* fabrication of autologous, living tissue with the potential for regeneration of heart muscle. The general concept of WJ-MSCs based cardiovascular tissue engineering has been validated in large animal studies [75]. In brief, completely autologous, living trileaflet heart valves generated using human WJ-MSCs have been successfully implanted in growing sheep models for up to 20 weeks. These valves showed good functional performance as well as structural and biomechanical characteristics strongly resembling those of native semilunar heart valves. In comparative studies of various cell sources for cardiovascular tissue engineering, UC stem cell represent an attractive, readily available autologous cell source for cardiovascular tissue engineering offering the additional benefits of utilizing juvenile cells and avoiding the invasive harvesting of intact vascular structures [6]. Recently, a 3D aligned microfibrous myocardial tissue construct cultured under transient perfusion was introduced [76]. The goal of this study was to design and develop a myocardial patch to use in the repair of myocardial infarctions or to slow down tissue damage and improve long-term heart function. The basic 3D construct design involved two biodegradable macroporous tubes, to allow transport of growth media to the cells within the construct, and cell seeded, aligned fiber mats wrapped around them. The microfibrous mat housed WJ-MSCs aligned in parallel to each other in a similar way to cell organization in native myocardium. The 3D construct was cultured in a microbioreactor by perfusing the growth media transiently through the macroporous tubing for 14 days. Experimental data confirmed that 3D constructs from static and perfused cultures enhanced cell viability, uniform cell distribution and alignment due to nutrient provision from inside the 3D structure. Experimental results during the last decade have shown that WJ-MSCs have great potential in tissue engineering, in which one of most promising directions is cardiovascular tissue engineering [72]. Despite knowledge of their advanced characteristics and first reports of successful pre-clinical and clinical applications, WJ-MSCs require further study to determine their clinical limitations and establish realistic clinical protocols. For example, replacements currently applicable in scaffold-based tissue engineering are mostly based on foreign materials, such as natural, synthetic or hybrid polymers. This results in a lack of growth and remodelling and carries the risk for thrombo-embolic complications and infections. Possible problems concerning these systems are systemic toxicity, growth limitation, differentiation and function restraints, incorporation barriers and cell or tissue delivery difficulties. Thus, the development of compatible biomaterials that do not mitigate WJMSC regenerative- and immuno-modulatory-potential is necessary [72]. In addition, because long term survival of the stem cells in the host tissue and establishment of treatment regimen are critical issues which still hamper broad clinical applications of WJ-MSCs, the establishment of relevant clinical criteria for isolation, characterization, long-term cultivation, and maintenance of human MSCs is necessary for the successful use of WJ-MSCs in regenerative medicine.

### 4.4. Cartilage Regeneration

Cartilage is a specialized connective tissue which has poor regeneration and self-repair capacity *in vivo*. Traumatic injury or autoimmune processes are among the main causes of cartilage damage and degeneration, for which new hope comes from tissue engineering using stem cells which have undergone chondrocyte-like differentiation. To this end, *in vitro* and *in vivo* data on the use of perinatal stem cells, in particular WJ-MSC, for regenerative medicine aimed at cartilage repair and regeneration have been reported [77]. UCMSCs are able to differentiate into chondrocyte-like cells if cultured in a supplemented medium. Analysis of the chondrogenic potential of WJ-MSCs showed they have the multipotential capacity and their chondrogenic capacity could be useful for future cell therapy in articular diseases [78]. Wang *et al*. demonstrated that seeding density of WJ-MSCs in poly-glycolic acid (PGA) scaffolds, in the presence of chondrogenic medium, had important effects on their chondrogenic potential [79]. This study demonstrated the potential for chondrogenic differentiation of WJ-MSCs in three-dimensional tissue engineering; higher seeding densities better promoted biosynthesis and mechanical integrity, and thus a seeding density of at least 25 million cells/mL is recommended for fibrocartilage tissue engineering with umbilical cord mesenchymal stromal cells [79]. Chondrogenic differentiation of WJ-MSCs can also be enhanced when cultured on nanofibrous substrates with a sequential two cultures medium environment. Moreover, WJ-MSCs are able to upregulate the production of hyaluronic acid and GAGs, as well as the expression of key genes as SOX9, COMP, Collagen type II and FMOD [80]. Because osteochondral tissue consists of cartilage and bone, cell sources and tissue integration between cartilage and bone regions are critical to successful osteochondral regeneration. Recently, Wang *et al*. developed a supportive structure which mimics native osteochondral tissue [81]. In this study, WJ-MSCs were introduced to the field of osteochondral tissue engineering and a new strategy for osteochondral integration was developed by sandwiching a layer of cells between chondrogenic and osteogenic constructs before suturing them together. Two groups of WJ-MSCs were seeded in different poly-l-lactic-acid (PLLA) scaffolds with chondrogenic and osteogenic medium respectively for 3 weeks. After this period of time, chondrogenic and osteogenic constructs were sutured together surgically to create four different osteochondral assemblies. Histological and immunohistochemical staining, such as for glycosaminoglycans, type I collagen and calcium, revealed better integration and transition of the matrices between two layers in the composite group containing sandwiched cells as compared to other control composites. These results suggest that hUCMSCs may be a suitable cell source for osteochondral regeneration, and the strategy of sandwiching cells between two layers may facilitate scaffold and tissue integration [81]. In short, WJ-derived cells are promising cellular source for cartilage repair due to both their differentiation and immunomodulatory properties. WJ-MSCs have been demonstrated to successfully differentiate into cells resembling mature chondrocytes. Moreover, their peculiar features of low innunogenicity and their potential to induce immune tolerance in the host justify the efforts for their use in osteoarthritis, rheumatoid arthritis and other disease settings. The high variability of cell sources, the need for scaffolds and matrixes, and the administration of several combinations of growth factors necessitates further research to optimize this cellular therapy approach and translate the results obtained from bench to clinic for cartilage repair.

### 4.5. Peripheral Nerve Repair

Many therapeutic approaches have been used in an attempt to restore neural function after PNS injury. Recent tissue engineering studies have focused on the development of bioartificial nerve conduits to guide axonal regrowth [82,83]. In this system, the bioartificial nerve conduit is placed between the nerve ends to enclose intervening gap, thereby allowing axons to regrow into the distal nerve segment. However, artificial nerve conduits are limited when the nerve gap is long. Schwann cells, one of the most important components of the peripheral glia that forms myelin, serve as a favorable microenvironment for the repair of damaged nerve fibers in the peripheral nervous system (PNS) [84]. As a rule, Schwann cells are crucial for PNS regeneration, even when artificial nerve conduits are used. Because isolation and expansion of Schwann cells from other peripheral nerve have limitations, many researchers have focused on MSCs from various types of tissues. The induction system for differentiating Schwann cells from BM-MSCs was first reported by Dezawa *et al*. in 2001 [85]. Recently, UCMSCs were shown to differentiate into Schwann cells capable of supporting neural regeneration and constructing myelin [86,87]. Transplantation into rat transected sciatic nerve showed that the human UC-Schwann cells maintained their differentiated phenotype *in vivo* after transplantation and contributed to axonal regeneration and functional recovery. Another group demonstrated that UC-Schwann cells differentiated from WJ produced neurotrophic factors such as NGF and BDNF [88,89]. These findings indicated that UC-Schwann cells are a viable alternative to native Schwann cells and may be applied to cell-based therapy for nerve injuries. Given the intrinsic ability of activated Schwann cells to promote axonal regeneration *in vivo*, UCMSC can be used to successfully derive mature Schwann cells for the regeneration of peripheral nerve. Schwann cells also support axonal regeneration, construct myelin, and contribute to functional recovery in a spinal cord injury model. In addition to WJ, Schwann cells can be differentiated from MSCs harvested from other sources, such as BMSCs, UC-MSCs, and ADSCs. In the end, a vis-à-vis comparison among these many MSC sources can reveal the potential of WJ-derived MSCs for therapeutic application to spinal cord injury [87].

Along this line of investigations, efforts to maximize the isolation and differentiation of stem cells derived from WJ have utilized studies designed to optimize cell harvest protocols, such as the use of oxygen concentration and plating density [90]. Such standardized isolation protocols would permit the expansion and maintenance of colony forming unit-fibroblast (CFU-F). Previous work reported that low plating density and/or exposure to 5% oxygen *vs*. 21% oxygen increased proliferation rate and enhanced expansion of MSCs. Recently, the effects of both plating density and oxygen concentration on MSCs derived from WJ have been evaluated [90]. Reducing oxygen concentration from 21% (room air) to 5% during expansion increased cell yield and maintained CFU-F, without affecting the expression of surface markers or the differentiation capacity of WJ-MSCs. The proposed mechanism is that reducing oxygen concentration in culture up-regulates hypoxia inducible factors (HIFs) and downstream effects from HIF activation include increased cell proliferation and maintenance of CFU-F, perhaps by affecting telomerase. In addition, reducing plating density from 100 to 10 cells/cm^2^ increased CFU-F frequency. Therefore, plating density and oxygen concentration are two important variables that affect the expansion rate and frequency of CFU-F of WJ-MSCs. These results suggest that these two variables are key stem cell isolation factors to produce different input populations for tissue engineering or cellular therapy.

### 4.6. Cardiac Differentiation of Human WJ-Derived Stem Cells

Since undifferentiated MSC tend to spontaneously differentiate into multiple lineages when transplanted *in vivo*, the developmental fate of transplanted BM-MSCs is not restricted by the surrounding tissue after myocardial infarction. It is possible that such uncommitted stem cells undergo maldifferentiation within the infracted myocardium with potentially life-threatening consequences [91]. Therefore, it was postulated that a certain cardiac differentiation of stem cells prior to transplantation would result in enhanced myocardial regeneration and recovery of heart function [92,93]. In this context, initiating the transformation of stem cells into a cardiomyogenic lineage is accomplished by culturing them in defined culture conditions. WJ-MSCs can be induced toward heart cells; after 5-azacytidine treatment for 3 weeks, WJCs expressed the cardiomyocyte markers, cardiac troponin I, connexin 43, and desmin, and exhibited cardiac myocyte morphology [94]. In addition, oxytocin, embryo-like aggregates and several growth factors like transforming growth factor-β1 (TGF-β1), PDGF and basic fibroblast growth factor (bFGF) are used to induce myocyte differentiation of various stem cell types [95–97]. The expression levels of oxytocin are higher in developing hearts than in adult hearts suggesting that oxytocin may be involved in cardiomyocyte differentiation [98]. A variety of protocols of cardiac differentiation designed for different stem cell types have been published [97]. One such study showed that cardiac differentiation of UCMSC was driven by cell treatment with 5-azacytidine, oxytocin as well as by forming of “embryoid bodies” [97]. The morphological and immunocytochemical analysis of cardiac differentiated UCMSC (cUCMSC) with an extensive panel of cardiac markers showed that oxytocin is a more potent inducer of cardiac differentiation than 5-azacytidine and the forming of “embryoid bodies”. In conclusion, comparative immunocytochemical analyses revealed that WJ-MSCs can be differentiated into cardiomyocyte-like cells with oxytocin being the most efficient differentiation agent. Very recently, a comparison study reported the long-term therapeutic effect of MSC from two different sources (adult bone marrow or Wharton’s jelly from umbilical cord) following MI in a rat model [99]. A significant improvement in ejection fraction was seen in animals that received MSCs in time points 25 to 31 wks after treatment. In addition, Wharton’s jelly MSCs were co-cultured with fetal or adult bone-derived marrow MSCs to investigate MSCs’ cardiac differentiation potential. When Wharton’s jelly MSCs were co-cultured with fetal MSCs, and not with adult MSCs, myotube structures were observed in two-three days and spontaneous contractions (beating) cells were observed in five-seven days. Taken together, these results suggest that MSCs administered 24–48 h after MI have a significant and a strong beneficial effect lasting longer than 25 weeks after MI; additionally, WJCs may be a useful source for off-the-shelf cellular therapy for MI.

The easy accessibility and the ability of UCMSC to differentiate into cells with characteristics of cardiomyocytes render UCMSC an attractive candidate for cell based therapies and cardiac tissue engineering. The next step is to show whether UCMSC, as well as WJ-derived stem cells, possess functional properties of cardiomyocytes in order to fully assess their utility for cardiac repair.

## 5. The New Research Frontiers in WJ Research

### 5.1. Clonal MSCs

A rich source of human MSCs was found in the perivascular region of the human UC which called HUCPVCs [24,100,101] which has enabled the first robust single cell clonal confirmation of a hierarchy of MCS differentiation [102]. The isolation of a nonhematopoietic (CD45−, CD34−, SH2+, Thy-1+, CD44+) HUCPVC population [24] may represent a significant source of cells for allogeneic MSC-based therapies due to their rapid doubling time, high frequencies of CFU-F and CFU-osteogenic subpopulation, and high MHC−/− phenotype. HUCPVCs show a similar immunological phenotype to bone marrow-derived MSCs (BM-MSCs) and present a non-hematopoietic myofibroblastic MSC phenotype (CD45−, CD34−, CD105+, CD73+, CD90+, CD44+, CD106+, 3G5+, CD146+) [103]. In addition to robust quinti-potential differentiation capacity *in vitro*, HUCPVCs have been shown to contribute to both musculo-skeletal and dermal wound healing *in vivo* [103]. Similar clonal expansions of WJ-derived stem cells will provide a well-defined set of stem cells allowing consistent validation and replication of studies that could enhance successful translation of laboratory studies of WJ for therapeutic applications.

### 5.2. Use of Magnetic Resonance Imaging in Contrast Labeled-UC Stem Cells

A recent study reported the isolation of cells from the intervascular and perivascular portion of UCM and compared these cell lineages by characterization of their specific marker expression patterns, capacity for self-renewal and potential to differentiate into multiple lineages [104]. The cells isolated from the intervascular portion showed faster doubling times than cells from the perivascular portion (which are probably more highly differentiated). Cells from both portions expressed MSC mRNA markers (CD29, CD105, CD44, CD166) and were negative for CD34 and MHC-II. Osteogenic, adipogenic, chondrogenic and neurogenic differentiation were confirmed by specific staining and gene expression. Another aim of this study was to investigate their labeling efficiency of MSC with magnetic resonance contrast agents. To investigate this, pre-clinical experiments involving labeling of cells with magnetic resonance contrast agents (superparamagnetic iron oxide particles-SPIO-and manganese chloride) and the subsequent *in vitro* study of these were conducted. Both contrast agents were found to provide simple, robust and safe methods to label cells; nevertheless, SPIO-labeling method has higher sensitivity. The SPIO labeling procedure proved to be an efficient and non-toxic tool that merits further investigation and the possible development of *in vivo* studies for clinical applications. Such studies will not only provide evidence of stem cell migration and deposition to injured and non-injured tissues, but will also offer insights on mechanisms of action of cell therapy.

## 6. Conclusions

Altogether, these studies offer authoritative views on phenotypic markers and therapeutic potential of WJ-derived stem cells. We provide insights on gaps in knowledge for the cells’ biological properties and translational applications. Cognizant of the many tissue sources of stem cells, further investigations on the advantages and limitations of WJ will reveal their optimal transplant regimens that are tailored for specific diseases.
